# Hemolytic Uremic Syndrome Risk and *Escherichia coli* O157:H7

**DOI:** 10.3201/eid1112.050607

**Published:** 2005-12

**Authors:** Boldtsetseg Tserenpuntsag, Hwa-Gan Chang, Perry F. Smith, Dale L. Morse

**Affiliations:** * University at Albany, Albany, New York, USA; † New York State Department of Health, Albany, New York, USA

**Keywords:** hemolytic uremic syndrome, Escherichia coli, risk factor, dispatch

## Abstract

We reviewed medical records of 238 hospitalized patients with *Escherichia coli* O157:H7 diarrhea to identify risk factors for progression to diarrhea-associated hemolytic uremic syndrome (HUS). Data indicated that young age, long duration of diarrhea, elevated leukocyte count, and proteinuria were associated with HUS.

In the United States, *Escherichia coli* O157:H7 causes ≈73,000 infections and 60 deaths annually (*1*). Infection progresses to hemolytic uremic syndrome (HUS) in 2% to 15% of cases (*2*). In studies of *E. coli* O157:H7 outbreaks, female sex, young age, elevated leukocyte count, antimicrobial drug use, vomiting, and fever have been reported as risk factors for HUS (*3**–**11*). Previously, a possible association between HUS and female sex, young age, and prolonged duration of diarrhea was shown in a study that evaluated the New York state surveillance system for postdiarrheal HUS (*12*). This report extends that study to investigate hospitalized patients with *E. coli* O157:H7 infection to assess potential risk factors for progression of infection to HUS by using a case-control study.

## The Study

Medical charts of all persons who were hospitalized and reported with confirmed cases of *E. coli* O157:H7 to the New York State Department of Health's Communicable Disease Surveillance System (CDSS) in 1998 and 1999 were reviewed according to a standardized survey form. A HUS case was defined as occurring in a patient with acute diarrhea who was hospitalized with *E. coli* O157:H7 infection and in whom confirmed or probable postdiarrheal HUS developed. A confirmed HUS case was defined as occurring in a patient with a clear history of acute diarrhea who showed the following signs: hemolytic anemia with microangiopathic changes, renal insufficiency (creatinine level >1.0 mg/dL in a child <13 years of age or >1.5 mg/dL in an adult, or >50% increase over baseline), and thrombocytopenia (platelet count <150,000/μL). A probable HUS case was defined as occurring in a patient with acute diarrhea with all the above signs except microangiopathic changes in the blood smear. Controls were hospitalized patients with *E. coli* O157:H7 infection without HUS. Demographic, clinical, and laboratory characteristics were abstracted from medical charts. Statistical analysis was performed by using SAS software (SAS Institute, Cary, NC, USA). A multiple logistic regression analysis was performed to identify factors associated with development of HUS.

In 1998 and 1999, the CDSS received reports of 1,170 cases of *E. coli* O157: H7 infection. Of these, 255 patients (21%) were hospitalized and 238 (93%) had medical charts available for review. Thirty-six (15%) patients were confirmed (n = 29) or probable (n = 7) HUS case-patients, and 202 *E. coli* O157:H7–infected patients without HUS were identified as controls. The risk of HUS was highest among children <5 years of age, compared with patients >65 years (odds ratio [OR] 4.9, 95% confidence interval [CI] 2.2–11.8). Sixty-nine percent of HUS patients were female compared with 61% of controls (OR 1.5, 95% CI 0.8–3.4). The hospital stay was significantly longer for HUS patients than controls (median hospital stay 13 vs. 3 days). Five HUS patients (14%) died, including 2 children <5 years of age, compared with 2 controls (1%).

Forty percent of all patients had vomiting, and 85% had bloody stool. These factors were not significantly different between patients and controls. Eleven (31%) case-patients and 78 (38%) controls were treated with antimicrobial drugs (not significant). Antimicrobial treatment was reported in 11 patients before the diagnosis of HUS: 6 received antimicrobial drugs primarily for other conditions (e.g., urinary tract infection, otitis media, venous line sepsis), 1 had treatment stopped once *E. coli* O157:H7 was diagnosed, and we could not tell whether drug regimens were completed or discontinued in 4 patients. HUS patients were more likely than non-HUS controls to have fever (OR 3.2, 95% CI 1.6–6.5). The duration of diarrhea before hospitalization was significantly longer for HUS patients than for non-HUS controls (median 4 vs. 2 days).

Proteinuria and hematuria were observed significantly more often among the case-patients. Twenty-three (64%) patients had proteinuria at admission, whereas 37 (18%) controls were admitted with proteinuria (OR 7.8, 95% CI 3.6–17). Hematuria at admission was reported in 23 (64%) patients and 57 (28%) controls (OR 4.5, 95% CI 2.1–9.4). Twenty-nine (81%) HUS patients vs. 90 (44%) controls had leukocyte counts >13,000/μL (OR 5.2, 95% CI 2.2–12.3) at admission (Table 1). Factors associated with HUS in univariate analysis (age <5 years, outbreak case, fever, hematuria, proteinuria, leukocytosis at admission, and duration of diarrhea before hospitalization >3 days) were included in the multivariate analysis. The following variables were associated with HUS development in the multivariate analysis: proteinuria (OR 6.7, 95% CI 1.9–24.1), duration of diarrhea before hospitalization >3 days (OR 6.2, 95% CI 2.2–17.4), age <5 years (OR 5.9, 95% CI 1.9–17.6), and leukocyte count >13,000/μL (OR 4.4, 95% CI 1.6–12.6). Factors such as outbreak involvement, hematuria and fever were not associated with HUS development (Table 2).

**Table 1:** Characteristics of hospitalized Escherichia coli O157:H7 patients by HUS case status, New York, 1998–1999*

Characteristic	Total (N = 238) n (%)	HUS (n = 36) n (%)	Non-HUS (n = 202) n (%)	OR (95% CI)	p value
Age (y)					
0–4	34 (14)	18 (49)	16 (8)	4.9 (2.2–11.8)	<0.001
5–14	52 (22)	6 (17)	46 (23)	1.1 (0.4–3.1)	
15–65	96 (24)	6 (17)	90 (44)	0.6 (0.2–1.7)	
>65	56 (40)	6 (17)	50 (25)	1.0	
Sex					
Female	147 (62)	25 (69)	122 (61)	1.5 (0.8–3.4)	0.33
Male	91 (38)	11 (31)	80 (39)	1.0	
Outcome					
Dead	7 (3)	5 (14)	2 (1)	16.1 (2.9–86.8)	0.001
Alive	231 (97)	31 (86)	200 (99)	1.0	
Outbreak					
Yes	49 (21)	15 (42)	34 (17)	3.6 (1.6–7.5)	0.01
No	189 (79)	21 (58)	168 (83)	1.0	
Hospital stay (d)					
>4	121 (51)	31 (86)	90 (45)	7.7 (2.8–20.6)	0.001
1–4	117 (49)	5 (14)	112 (55)	1.0	
Bloody stool					
Yes	203 (85)	30 (84)	173 (86)	0.8 (0.3–2.4)	0.77
No	35 (15)	6 (16)	29 (14)	1.0	
Fever					
Yes	71 (30)	19 (53)	52 (26)	3.2 (1.6–6.5)	0.009
No	167 (70)	17 (47)	150 (74)	1.0	
Vomiting					
Yes	96 (40)	14 (39)	82 (40)	0.9 (0.4–1.9)	0.84
No	142 (60)	22 (61)	120 (60)	1.0	
Antimicrobial drug use					
Yes	89 (37)	11 (31)	78 (38)	0.7 (0.3–1.5)	0.38
No	149 (63)	25 (69)	124 (62)	1.0	
Proteinuria at admission					
Yes	60 (25)	23 (64)	37 (18)	7.8 (3.6–17.0)	<0.001
No	178 (75)	13 (36)	165 (82)	1.0	
Hematuria at admission					
Yes	80 (34)	23 (64)	57 (28)	4.5 (2.1–9.4)	<0.001
No	158 (66)	13 (36)	145 (72)	1.0	
Leukocyte count at admission					
>13,000/μL	119 (50)	29 (81)	90 (44)	5.2 (2.2–12.3)	<0.001
<13,000/μL	119 (50)	7 (19)	112 (56)	1.0	
Duration of diarrhea before hospitalization					
>3 days	70 (29)	24 (67)	46 (23)	6.7 (3.1–14.6)	<0.001
<3 days	168 (71)	12 (33)	156 (77)	1.0	

*HUS, hemolytic uremic syndrome; OR, odds ratio; CI, confidence interval.

**Table 2:** Multiple logistic regression analysis of risk factors associated with HUS, New York, 1998–1999*

Characteristic	No. patients (%) (n = 36)	No. controls (%) (n = 202)	Adjusted OR (95% CI)
Proteinuria	23 (64)	37 (18)	6.7 (1.9–24.1)
Duration of diarrhea before hospitalization >3 d	24 (67)	46 (23)	6.2 (2.2–17.4)
Age <5 y	18 (50)	16 ( 8)	5.9 (1.9–17.6)
Leukocytes >13,000/μL	29 (81)	90 (44)	4.4 (1.6–12.6)
Outbreak case	15 (42)	34 (17)	1.7 (0.6–4.9)
Hematuria	23 (64)	57 (28)	1.4 (0.4–4.9)
Fever	19 (53)	52 (26)	1.1 (0.4–3.1)

*HUS, hemolytic uremic syndrome; OR, odds ratio; CI, confidence interval.

## Conclusions

This study provides additional information on potential risk factors for progression of *E. coli* O157:H7 infection to HUS, but unlike other studies, this study used hospitalized rather than outpatient controls. Our data confirmed previous differences in risk for HUS development by age group (*3**–**5*). Women and girls have been reported to be at increased risk for HUS development in several studies (*10**,**11*), but our study showed no significant increased risk. Several studies have suggested that administration of antimicrobial agents increases risk for HUS development (*5**,**6**,**9**,**13*), but no significant relationship was observed between HUS and the use of antimicrobial drugs in our sample.

Although reports (*5**,**7*) have demonstrated a higher incidence of HUS among patients with bloody diarrhea, fever, or vomiting, our multivariate analysis did not show a significant association between these characteristics and HUS. Since only hospitalized patients with severe diarrhea were studied, some symptoms (bloody stool, fever, or vomiting) might have been reported more often than in the general population with *E. coli* O157:H7 infection. As a result, some significant associations might have been missed. Buteau et al. (*14*) reported that a diarrheal prodrome <3 days is an independent predictor of HUS development in children with *E. coli* O157:H7 infection; however, our study suggested that prolonged diarrhea (>3 days) may increase the risk of HUS.

Our analysis was consistent with results of other studies that found patients with elevated leukocyte counts to be at higher risk for developing HUS (*5**–**8**,**14*). Patients with leukocytes >13,000/μL at admission in our study had 5 times the risk of HUS. Protein and occult blood in urine were described as risk factors for HUS in a study in Japan (*15*). In the current study, proteinuria at admission was also a risk factor for HUS. However, HUS had already developed in most of these patients by the time of hospitalization, and we could not determine whether these factors preceded HUS development.

In summary, patients hospitalized for *E. coli* O157:H7 infection, those <5 years of age with >3 days of diarrhea, leukocytes >13,000/μL, and proteinuria should be monitored closely for further complications. Nine (25%) of the HUS patients had 4 risk factors, 11 (31%) patients had 3 risk factors, and 10 (28%) had 2 risk factors. In comparison, none of the controls had these 4 risk factors, 4 (2%) had 3 risk factors, and 47 (23%) had 2 risk factors. Identifying potential risk factors may allow clinicians to develop treatment interventions to prevent progression to HUS.
